# Genome-wide high-throughput signal peptide screening via plasmid pUC256E improves protease secretion in *Lactiplantibacillus plantarum* and *Pediococcus acidilactici*

**DOI:** 10.1186/s12864-022-08292-3

**Published:** 2022-01-12

**Authors:** Binbin Chen, Bryan Zong Lin Loo, Ying Ying Cheng, Peng Song, Huan Fan, Oleg Latypov, Sandra Kittelmann

**Affiliations:** 1 Wilmar International Limited, WIL@NUS Corporate Laboratory, Centre for Translational Medicine, National University of Singapore, Singapore, Singapore; 2 Wilmar International Limited, Wilmar (Shanghai) Biotechnology Research and Development Center Co. Ltd., Shanghai, China; 3 Present Address: Huan Fan, Center for Integrative Conservation, Xishuangbanna Tropical Botanical Garden, Chinese Academy of Sciences, Menglun, Yunnan People’s Republic of China

**Keywords:** Signal peptide, Protease secretion, High-throughput screening, Genetic engineering, *Lactiplantibacillus plantarum*, *Pediococcus acidilactici*

## Abstract

**Background:**

Proteases catalyze the hydrolysis of peptide bonds of proteins, thereby improving dietary protein digestibility, nutrient availability, as well as flavor and texture of fermented food and feed products. The lactobacilli *Lactiplantibacillus plantarum* (formerly *Lactobacillus plantarum*) and *Pediococcus acidilactici* are widely used in food and feed fermentations due to their broad metabolic capabilities and safe use. However, extracellular protease activity in these two species is low. Here, we optimized protease expression and secretion in *L. plantarum* and *P. acidilactici* via a genetic engineering strategy.

**Results:**

To this end, we first developed a versatile and stable plasmid, pUC256E, which can propagate in both *L. plantarum* and *P. acidilactici*. We then confirmed expression and secretion of protease PepG1 as a functional enzyme in both strains with the aid of the previously described *L. plantarum*-derived signal peptide LP_0373. To further increase secretion of PepG1, we carried out a genome-wide experimental screening of signal peptide functionality. A total of 155 predicted signal peptides originating from *L. plantarum* and 110 predicted signal peptides from *P. acidilactici* were expressed and screened for extracellular proteolytic activity in the two different strains, respectively. We identified 12 *L. plantarum* signal peptides and eight *P. acidilactici* signal peptides that resulted in improved yield of secreted PepG1. No significant correlation was found between signal peptide sequence properties and its performance with PepG1.

**Conclusion:**

The vector developed here provides a powerful tool for rapid experimental screening of signal peptides in both *L. plantarum* and *P. acidilactici*. Moreover, the set of novel signal peptides identified was widely distributed across strains of the same species and even across some closely related species. This indicates their potential applicability also for the secretion of other proteins of interest in other *L. plantarum* or *P. acidilactici* host strains. Our findings demonstrate that screening a library of homologous signal peptides is an attractive strategy to identify the optimal signal peptide for the target protein, resulting in improved protein export.

**Supplementary Information:**

The online version contains supplementary material available at 10.1186/s12864-022-08292-3.

## Background

The lactobacilli (or family Lactobacillaceae until 2020) are a highly diverse group of lactic acid-producing bacteria. Species within this group were formerly classified into only three genera, *Lactobacillus*, *Paralactobacillus*, and *Pediococcus*, and were only recently re-classified into 26 different genera, including the genera *Lactiplantibacillus* (formerly *Lactobacillus*) and *Pediococcus* [1]. They can be found in many ecological niches, such as on living and decaying plant material, as well as in naturally fermented meat, vegetables, milk and silages [2, 3]. Colonization of the digestive tract of mammalian hosts by members of the lactobacilli is also frequently observed [4, 5]. Some species of lactobacilli are “generally recognized as safe”, and these are some of the economically most important species as they are routinely used in a variety of industrial food and feed fermentations [6]. Many beneficial effects for human and animal health have been attributed to these species, some of which are supported by a large body of scientific literature, e.g., elimination of pathogens through lactic acid and bacteriocin production [7, 8], production of beneficial metabolites and vitamins [9], reduction of cholesterol [10], antioxidant activity [11], as well as a broad range of other health promoting and disease preventing effects [12, 13]. Moreover, fermented food and feed are generally characterized by an enhanced texture, flavor, aroma and nutritional value, due to the abundance and diversity of secreted metabolites (e.g., organic acids, ketones, and aldehydes) and enzymes (e.g., amylases, esterases, glucosidases, lipases, and proteases) [14, 15]. Proteases have been intensively studied in lactobacilli [16–19]. Proteases catalyze the hydrolysis of peptide bonds of proteins that are present in complex food and feed matrices. This process results in the release of peptides and free amino acids essential for cell growth. Hence, protease activity is particularly important to those species auxotrophic for amino acids, which often occur in milk fermentations [17]. Proteolytic activity improves dietary protein digestibility and nutrient utilization by increasing the relative amount of small peptides [20]. Moreover, proteases break down allergenic proteins and trypsin inhibitors, e.g., in soybean-derived substrates, which results in improved acceptance and higher uptake especially by monogastric animals [21]. In addition, proteases contribute to flavor and texture of fermented products [18]. For these reasons, investigations into the diversity and activity of native proteolytic enzymes in lactic acid bacteria has been a focal point of research for several decades [18]. However, most species harbor cell envelope-associated proteinase, and its attachment to the cell wall limits the amount of protease produced [17, 18]. *Lactiplantibacillus plantarum* and *Pediococcus acidilactici* are two of the industrially most important species in food and feed fermentation [2]. Several studies have explored the possibility of improving enzyme activity via genetic engineering using these two species as models [22]. One of the most critical parameters to determine if secretion of a desired target protein will be successful or not is the capacity of the signal peptide used to transport the protein into the extracellular space [23]. So far, engineered secretion in *L. plantarum* and *P. acidilactici* has mostly been achieved via heterologous signal peptides, e.g., sslipA of *Bacillus subtilis* [24], M6 of *Streptococcus pyogenes* [25] and Usp45 of *Lactococcus lactis* [26]. Only a limited number of studies have focused on the identification of homologous signal peptides in *L. plantarum* [27], and, to the best of our knowledge, none are available for *P. acidilactici* yet. Native signal peptides, however, have been shown to lead to similar or higher secretion than constructs with heterologous signal peptides [28]. It is conceivable that native signal peptides are best recognized by the native secretory machinery of the host. One key problem in selecting suitable signal peptides is the difficulty in predicting their efficiency based on primary sequence information alone. In this study, we carried out a genome-wide analysis of signal peptides from *L. plantarum* and *P. acidilactici*. Predicted native signal peptides were then assessed in *L. plantarum* or *P. acidilactici* host strain for their capacity in directing secretion of heterologous protease PepG 1[29]. Several novel native signal peptides were identified that resulted in recombinant strains with improved protease secretion. Use of these strains may increase extracellular protein degradation and peptide content in food and feed matrices.

## Results

### Plasmid optimization

In recent years, numerous plasmid vectors have been constructed for members of the former genus *Lactobacillus* [30–32]. However, advanced cloning vectors with high transformation efficiency and structure stability in *E. coli*, *Lactiplantibacillus* (*Lactobacillus*) *plantarum* and *Pediococcus acidilactici* are still lacking. In order to construct a shuttle vector, an *E. coli* replicon, an *E. coli* selection marker, a lactobacilli replicon and a lactobacilli selection marker are required.

**Fig. 1:**
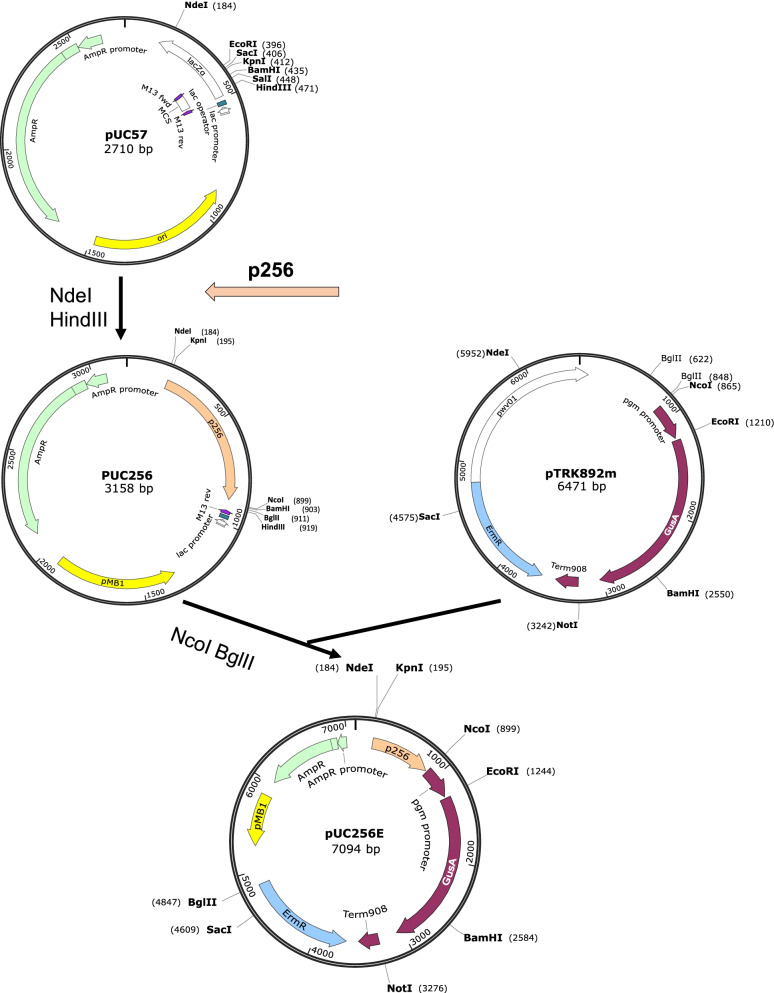
Schematic drawing of the construction of shuttle vector pUC256E. AmpR: ampicillin resistance marker; ErmR: erythromycin resistance marker; All restriction sites shown are unique, except for BglII in the pTRK892m

pUC57 was selected for its *E. coli* replicon and antibiotic resistance gene. In lactic acid bacteria, the most common replication mechanisms are the rolling circle and theta modes of replication [31]. Rolling circle mode of replicons, pSH71 [33], pWV01 [32, 34] and pLAB1000 [35], and theta type of replicons, pAmβ1 [30] and p256 [36] were selected. Among all five replicons, pSH71 and pWV01 suffered from structural instability during cloning in *E. coli*, as demonstrated by reduced plasmid size (data not shown), which is consistent with other reports [31]. Among pAmβ1, pLAB1000 and p256, only p256 showed successful expression of GusA in both *L. plantarum* and *P. acidilactici*, while pAmβ1 and pLAB1000 only showed positive colonies in *L. plantarum* or *P. acidilactici*, respectively. Thus, p256 was ligated into the MCS site of pUC57. Then, a 2948 bp fragment carrying the erythromycin selection marker (Erm^R^) and reporter gene GusA from pTRK892m was ligated to pUC256, resulting in shuttle vector pUC256E (Fig. 1). GusA was used to assess the potential of the expression system in lactobacilli. Upon expression, clear 5-bromo-4-chloro-3-indolyl glucuronide (X-Gluc) changes to a blue color. The appearance of blue-colored colonies on the agar suggested that pUC256E successfully expressed GusA. Successful plasmid construction was confirmed by sequencing. Subsequently, pUC256E was used for protease expression and signal peptide screening in *L. plantarum* and *P. acidilactici*.

### Protease expression and secretion in *L. plantarum* and *P. acidilactici*

**Fig. 2:**
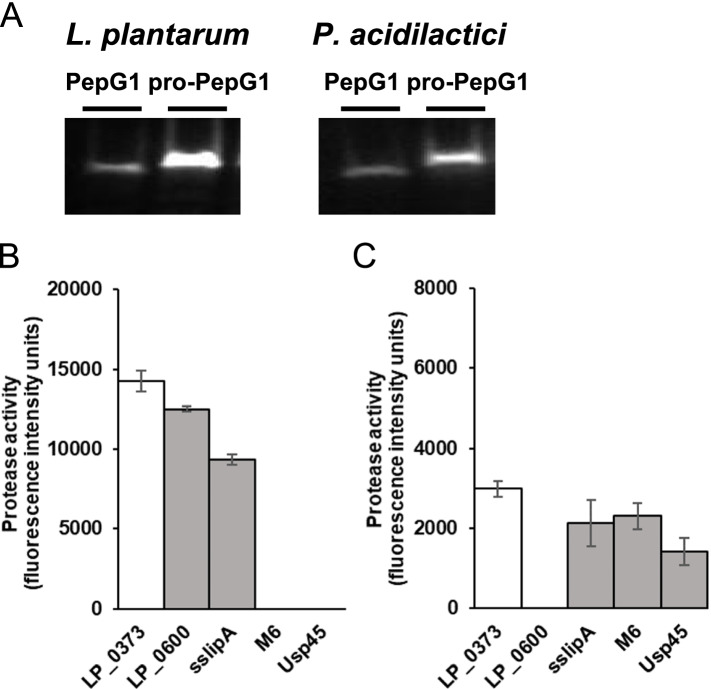
Protease expression and secretion in *L. plantarum* and *P. acidilactici*. **A** PepG1 and pro-PepG1 (PepG1 with propeptide) were expressed and secreted in both *L. plantarum* and *P. acidilactici* confirmed by western blot analysis. The protein bands corresponded to a molecular mass around 23 kDa, as deduced from positions of molecular weight standards (Bio-Rad; Precision Plus Protein Standards, not shown) and thus corresponded to mature PepG1. The protein bands of pro-PepG1 were visualized at slightly higher position on the gel due to the presence of the 2.6 kDa propeptide. For clarity and conciseness, blots of PepG1 and pro-PepG1 of *L. plantarum* were cropped from the image of the same gel, while blots of PepG1 and pro-PepG1 of *P. acidilactici* were cropped from the image of a second gel. The two blots were processed in parallel with the same exposure time (60 s). Full-length blots are presented in Supplementary Fig. S1. Protease activities in supernatants of *L. plantarum* (**B**) and *P. acidilactici* (**C**) harboring plasmids with different heterologous signal peptides. The white bars represent LP_0373, the control signal peptide chosen in this study. Enzyme activities are expressed in fluorescence intensity units. All results represent the means of three independent experiments; the error bars indicate the standard deviation (SD)

Lactobacilli are known for their production of lactic acid during growth, which leads to a lowered pH of the culture media. Five proteases, NprB from *Bacillus subtilis* (GenBank accession number: CAB01832.1), PepJ from *Aspergillus nidulans* (UniProtKB accession number: Q5AUR8.1), PepG1 from *Alicyclobacillus* sp. (GenBank accession number: ADG26771.1), PepA from *Aspergillus awamori* (PepA-Aa) (GenBank accession number: AAA78947.1) and PepA from *Aspergillus niger* (PepA-An) (GenBank accession number: CAK42031.1), were chosen based on the fact that those proteases show optimum protease activity at low pH, mainly between pH 4-5 [29, 37–40]. However, in order to hydrolyse the proteinaceous substrate in the media, a signal peptide is needed to initiate protease secretion. Here, a widely known *L. plantarum* signal peptide LP_0373, the best-performing native signal peptide of *L. plantarum* WCFS1 for secretion of model proteins NucA and AmyA, was selected as a benchmark [28]. To construct plasmids carrying these proteases, the ribosome binding site AGGAGG, signal peptide LP_0373 and respective protease were cloned into pUC256E by replacing the GusA coding sequence. The remaining pgm promoter at the 5′ end and Term 908 terminator at 3′ end of GusA were utilized as promoter and terminator for protease expression. Some studies have demonstrated that the fusion of a propeptide in-between the signal peptide and the mature moiety can enhance protein secretion [41, 42]. Therefore, in this experiment, we tested the effect of a propeptide by comparing protease secretion of constructs with and without the native propeptide in front of the mature protein. Proteins in supernatant and intracellular proteins were extracted and analysed by western blotting. Among all ten tested proteases, only PepG1 with and without the propeptide sequence (25.9 kDa, 23.3 kDa respectively) could be expressed and secreted in both *L. plantarum* and *P. acidilactici* (Fig. 2A). Only PepG1 without propeptide gave protease activity in both *L. plantarum* and *P. acidilactici*, therefore, PepG1 was chosen for further screening.

To increase protease secretion, we tested extracellular protease activity of four other well-known heterologous signal peptides, i.e. LP_0600 from *L. plantarum* [28], sslipA from *B. subtilis* [24], M6 from *Streptococcus pyogenes* [25] and Usp45 from *Lactococcus lactis* [26] in both *L. plantarum* (Fig. 2B) and *P. acidilactici* (Fig. 2C). All four signal peptides resulted in lower protease activity compared to LP_0373 in both *L. plantarum* and *P. acidilactici*. Therefore, homologous signal peptide screening was performed to increase protease secretion efficiency.

### Library construction

Secretion performance of signal peptides strongly depends on the expression host and target protein [25, 26]. Therefore, in this study, genomic scale prediction of native signal peptides was performed for our selected host strains. Native signal peptides of *L. plantarum* and *P. acidilactici* were predicted by SignalP using their proteome sequences as input. SignalP is a web-based program, which uses a deep neural network-based method incorporating conditional random field classification and improved transfer learning for optimized signal peptide prediction [43]. A total of 155 and 110 potential signal peptides were identified in *L. plantarum* and *P. acidilactici*, respectively. The length of the predicted signal peptides varied from ten to 49 amino acids for *L. plantarum* (30.0 ± 6.7 amino acids), among which LP_25440 was the shortest, and LP_23420 and LP_02480 were the longest. For *P. acidilactici*, the length of signal peptides varied from 16 to 52 amino acids (30.8 ± 7.7 amino acids), with PA_02840 being the shortest and PA_13520 being the longest. Bacterial signal peptides tend to have a prevalence of alanine at positions − 3 and − 1 relative to the cleavage site, giving rise to the name of the motif, Ala-X-Ala [44–46]. A total of 53 (out of 155) *L. plantarum* signal peptides have the consensus Ala-X-Ala cleavage site, while 32 (out of 110) *P. acidilactici* signal peptides contain the Ala-X-Ala cleavage site.

The respective signal peptides were fused to the N-terminal of PepG1 gene and downstream of pgm promoter. At the C-terminal of the signal peptide, two amino acids downstream of the predicted cleavage site were retained from the original protein. The cloning work was performed in *E. coli* cells. After transformation, selected colonies were sent for sequencing to confirm the diversity of the secretion tags in plasmids. Subsequently, over 2000 *E. coli* colonies were washed out and plasmids were extracted. After the preparation of the plasmid library, the cell libraries were created by transforming a mixture of the respective plasmids into *L. plantarum* or *P. acidilactici* cells. Notably, as opposed to *P. acidilactici*, transformation efficiency was poor for *L. plantarum* with the plasmids extracted from DH5α. Genome analysis revealed that *L. plantarum* SH LP contains a type IV restriction modification (R-M) system, which degrades methylated foreign DNAs. R-M systems in bacteria act as important defence mechanisms against invading genomes [47, 48]. To solve this issue, *E. coli* C2925 was chosen for unmethylated plasmid preparation, resulting in at least 1000-fold improved transformation efficiency of *L. plantarum*.

### Screening of signal peptides for improved PepG1 secretion capacity in *L. plantarum*

After confirming signal peptide diversity in transformants, a total of 1630 *L. plantarum* colonies (> 10-fold oversampling) were obtained and picked for screening. The strain harboring the expression vector with the LP_0373 signal peptide was used as the control. Protease activity was determined from the collected culture supernatants. A total of eighteen 96-well plates were screened to assess protease activity of all colonies. We shortlisted 126 colonies that showed ≥20% improved secretion capacity over the control signal peptide. Their plasmids were isolated and sequenced to determine the present signal peptides. Based on the sequencing results (Supplementary Table S1), a total of 12 different signal peptides were identified as potential candidates for improving the secretory expression of PepG1 in *L. plantarum* (Table 1).

**Table 1:** Characteristics of the signal peptides identified in the screening

Signal peptide	Amino acid sequence (putative cleavage site indicated by arrow)	Predicted function for corresponding protein	Length (amino acid)	Net charge of N-domain	Hydrophobicity (%)	Ala-X-Ala motif	Transmembrane helix
**Signal peptide with protease secretion**							
LP_23790	MKKFNFKTMLLLVLASCVFGVVVNVTTSLGPQTTITAQA↓SK	transglycosylase	39	3	59	✓	✓
LP_08330	MIKLRQVLKKILIVLMVFVLVFTAFSSSVDTVSA↓HR	hypothetical protein	34	4	65		✓
LP_04240	MKKLMCLFGVIGGLVFMSWTSPSIQATA↓TN	cell surface protein	28	2	68	✓	✓
LP_23670	MQLLKRIMVIVGTLILGLQVSSVSGLA↓AS	cell surface protein	27	2	70		✓
LP_28190	MKRLRHIKLGMLLLSCLAFISMLAITSQA↓AA	extracellular protein	29	4	62		✓
LP_29340	MRKWQVAVVMLLAALGSWFAIGTQAQA↓KT	glutamine ABC transporter substrate binding, permease	27	2	74	✓	✓
LP_23680	MPNKWWRLILGVMLVLSWAIPVRA↓AT	cell surface protein	24	2	79		✓
LP_28170	MKKMMRWLGAILVMISGLSAVVPAQA↓AN	cell surface protein	26	3	77	✓	✓
LP_23160	MQKRLRLSLGMLLAVVASLLMMGQVASA↓DQ	hypothetical protein	28	3	71	✓	✓
LP_24320	MRFAGKLKKVMIALVAAVTFSTAGLGIAGADLQAQA↓AS	D-alanyl-D-alanine carboxypeptidase	36	4	72	✓	✓
LP_14210	MKKIVNWLLGSVLMIAAVTMLSSVSANA↓ST	hypothetical protein	28	2	68	✓	✓
LP_09710	MRRLLTGTLVVGGLLLVVCLMAVNGQA↓KV	extracellular protein	27	2	74		✓
PA_18600	MVKSRNRILHYILVAVSVVIVVLGFSVIKASA↓HG	chitin-binding protein	32	3	66	✓	✓
PA_13510	MYKGFKKYFSNGADRKAGNYPVAKRNKRWLLASAVMLAMFGAGMAQSHAFA↓KA	hypothetical protein	51	9	59	✓	✓
PA_18250	MKLKAKLLLVVVPFLMGSVVYHPTPTVQA↓KT	DNA-entry nuclease	29	3	69		✓
PA_08950	MNQNWQKPSPKLNWVRFYSIVTILVLVTSVAGLEMLRVSA↓HQ	beta-lactamase class A	40	3	58		✓
PA_17320	MKKARWKLLLAGLALLGGISLGQNIISA↓NT	hypothetical protein	28	4	71		✓
PA_10610	MKRKWFSLLVAVFLIIGVAIGFGGILHSKSSG↓ND	hypothetical protein	32	3	72		✓
PA_04150	MKKAITTASFFLAIFVVFMVGSNAASA↓KS	hypothetical protein	27	2	70	✓	✓
PA_07000	METKKRFKMYKSGKKWLVAAIVAGGIATAGSVASVNA↓DE	hypothetical protein	38	7	59		✓
**Signal peptide without protease secretion**							
LP_27290	MRRKLVGYMLSMLTVILALFMLGSTAHA↓KE	cell surface protein	28	3	68	✓	✓
LP_27220	MKKINKLMILGMLVLGVTGATMINPEMTTA↓AH	extracellular protein	30	3	67		✓
LP_17340	MKKRFGWFLAIIVALIMTVVPLGQTQHAQA↓AD	ABC transporter substrate-binding and permease protein	30	3	70	✓	✓
LP_11950	MTKRMSFKFKWVALVATLIVGIGSWQVLAHA↓DS	hypothetical protein	31	4	68	✓	✓
LP_12630	MLKLIKQRLVWGLVLTATVSGVLSCNVAAHA↓TS	D-alanyl-D-alanine carboxypeptidase	31	3	65	✓	✓
LP_28330	MKLSKRGLFWLLGLVSFAILLLFSQPLGAQA↓AT	cell surface protein	31	3	74	✓	✓
LP_27010	MRKLIKACGLMVISMLVGLGIVTSALA↓AK	cell surface protein, CscB family	27	3	74	✓	✓
PA_14540	MKNNKIIITAAIAGLLGGGVAYGGASFVQNRMEA↓TT	serine protease	34	2	68		✓
PA_15330	MNYRSILFTTAIATMGAFSFGHSPVSA↓HS	hydrolase	27	1	59		
**Average (with protease secretion)**			31.5	3.4	68.2	50% with motif	100% with helix
**Average (without protease secretion)**			29.9	2.8	68.1	67% with motif	89% with helix
**t-test (with and without protease secretion)**			0.34	0.25	0.99	–	–

**Fig. 3:**
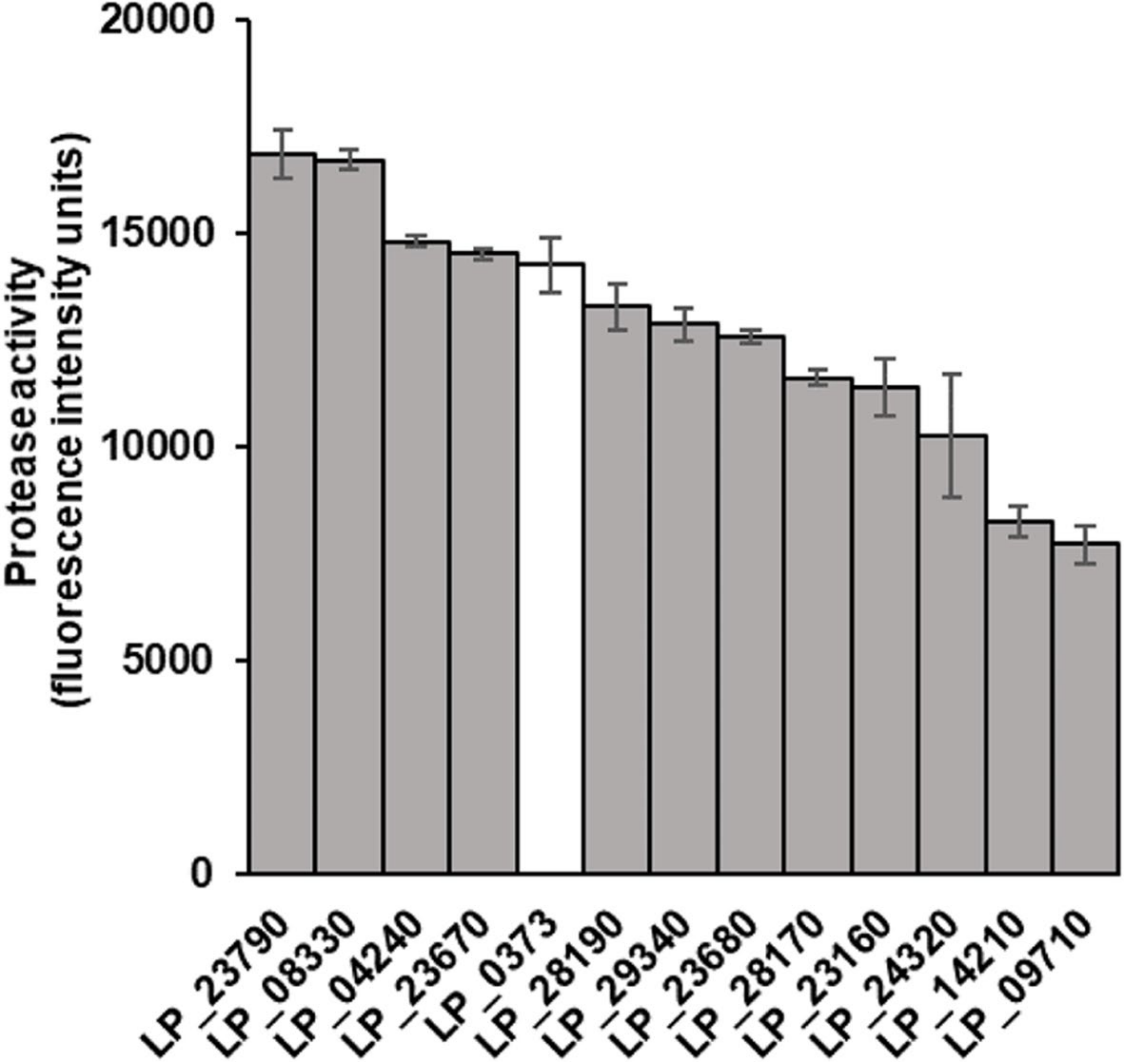
Secretion capacity of PepG1 of homologous signal peptides in recombinant *L. plantarum*. Only secretion capacities of the 12 homologous signal peptides with the highest PepG1 secretion capacities in *L. plantarum* are shown. The white bar represents LP_0373, which was chosen as the benchmarking signal peptide in this study. Data shown represent the mean ± SD of three biological replicates

To confirm the superior capacity of the signal peptides selected from the first round of screening on the PepG1 secretion level in *L. plantarum*, we re-evaluated the cells containing these signal peptides in triplicate. The cells containing LP_23790 and LP_08330 showed the highest secretion capacity. Their secretion efficiency was 18 and 17% higher than that of LP_0373 respectively, with *p*-value < 0.05 (Student’s *t*-test; Fig. 3). Compared to LP_0373, both LP_04240 and LP_23670 showed a higher secretion on average, however, the increase was not significant. The remaining seven signal peptides had lower capacities than LP_0373 (Fig. 3).

### Screening of signal peptides for improved PepG1 secretion capacity in *P. acidilactici*

The screening process for secretion capacity of PepG1 in *P. acidilactici* with its homologous signal peptide library was similar to that used for *L. plantarum*, and the same control, LP_0373, was used. A total of 1179 clones (> 10-fold oversampling) were selected and screened for protease activity. Out of these, 44 clones showed ≥50% improved protease activity and were sent for DNA sequencing to deduce the signal peptide sequences (Supplementary Table S1). A total of eight signal peptide sequences were retrieved, and cells carrying these were subjected to a second round of screening (Table 1).

**Fig. 4:**
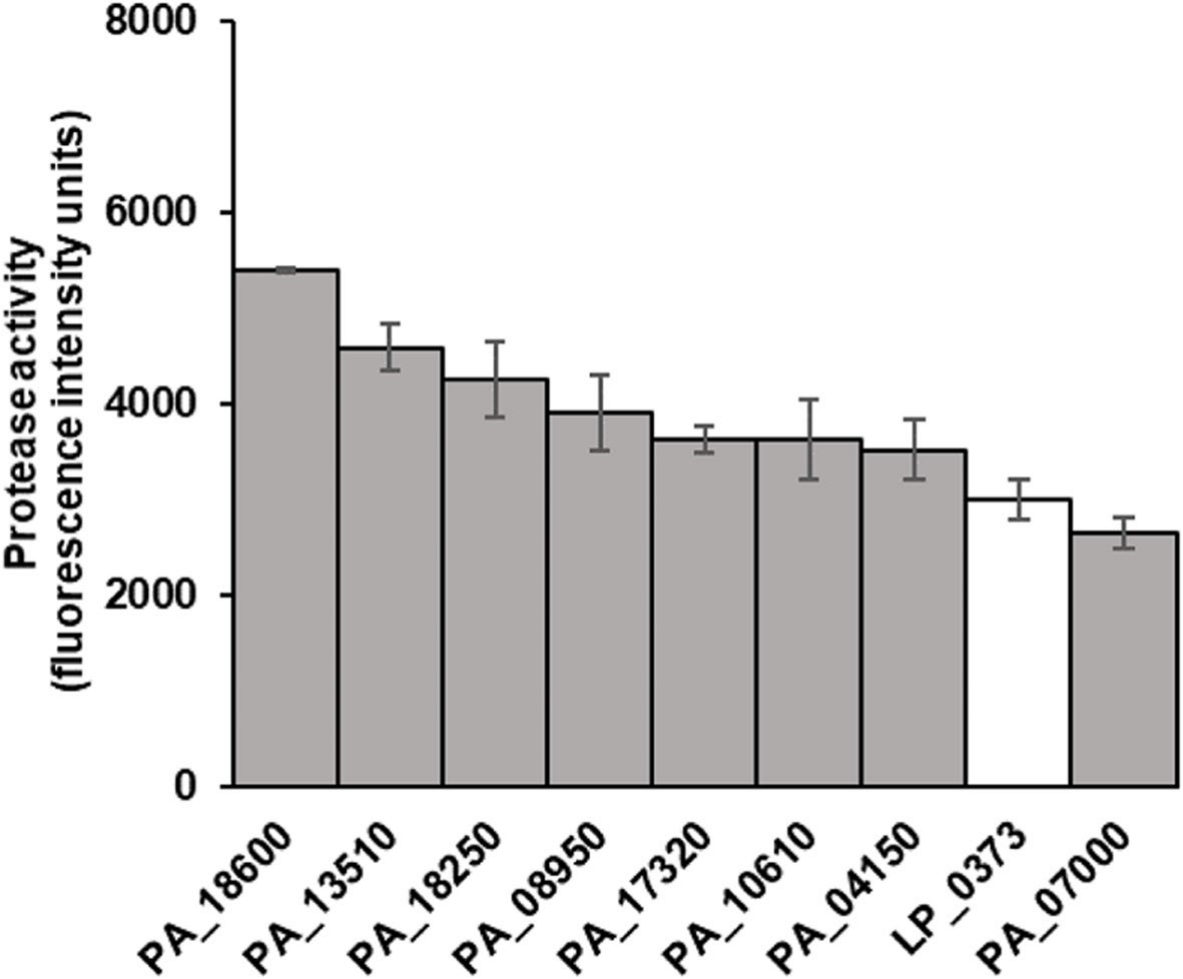
Secretion capacity of PepG1 of homologous signal peptides in recombinant *P. acidilactici*. Only secretion capacities of the 8 homologous signal peptides with the highest PepG1 secretion capacities in *P. acidilactici* are shown. The white bar represents LP_0373, which was chosen as the benchmarking signal peptide in this study. Data shown represent the mean ± SD of three biological replicates

Except for PA_07000, all homologous signal peptides achieved significantly higher secretion capacity than heterologous LP_0373 (*p* < 0.05, Student’s *t*-test). PA_18600 showed the highest secretion capacity, which was 80% higher than that of the control LP_0373 (Fig. 4).

### Correlations between signal peptide properties and measured secretion capacity for PepG1 protease

To further explore the potential correlation between the properties of the best-performing signal peptides and their high secretion capacity, we compared the 20 signal peptides with the highest protease secretion with nine signal peptides with no secretion activity identified during the screening process. It is known that signal peptides consist of three specific domains: a positively charged N-domain, a hydrophobic H-domain, and a neutral but polar C-domain containing the three amino acids which form the signal peptidase recognition site [27]. Here, several different properties of signal peptides were selected for analysis, namely, length, net charge of the N-domain, hydrophobicity, presence of an Ala-X-Ala cleavage site and transmembrane helix structure (Table 1).

Statistical analysis did not result in any significant differences between the two groups of signal peptides. These results are consistent with other studies [27, 49] which have shown that for specific protein, evaluation of signal peptide performance based on its amino acid sequence only proved to be an uphill task.

### Pertinence of identified active signal peptides in other strains and similarity of predicted signal peptides within species and genera

To understand the distribution and potential applicability of our identified active signal peptides, we assessed their presence in other strains and phylogenetically closely related species. Nine out of 12 identified *L. plantarum* signal peptides were present in more than half of the analyzed *L. plantarum* genomes, while the *P. acidilactici* signal peptides were detected in less than 60.9% of the analyzed *P. acidilactici* genomes (Table 2). In addition, seven out of 12 *L. plantarum* signal peptides appeared in other *Lactiplantibacillus* species, while none of the *P. acidilactici* signal peptides were present in other *Pediococcus* species (Table 2). It appears that the signal peptides identified in *L. plantarum* SH LP are more widely distributed than those identified in *P. acidilactici* SH PA.

**Table 2:** The presence of the identified signal peptides in other strains within the same species or the same genus

Signal peptide	Presence of signal peptide in 156 strains within ***L. plantarum*** species or 23 strains within ***P. acidilactici*** species^a^ (%)	Presence of signal peptide in 3 strains of other ***Lactiplantibacillus*** species or 4 strains of other ***Pediococcus*** species^a^ (%)
LP_23790	71.8	33.3
LP_08330	1.9	0.0
LP_04240	3.8	33.3
LP_23670	96.8	33.3
LP_28190	83.3	0.0
LP_29340	85.9	33.3
LP_23680	91.0	33.3
LP_28170	36.5	0.0
LP_23160	52.6	33.3
LP_24320	96.8	33.3
LP_14210	85.3	0.0
LP_09710	93.6	0.0
PA_18600	47.8	0.0
PA_13510	13.0	0.0
PA_18250	60.9	0.0
PA_08950	52.2	0.0
PA_17320	21.7	0.0
PA_10610	34.8	0.0
PA_04150	26.1	0.0
PA_07000	0.0	0.0

^a^These are the only strains that have complete genome sequences in NCBI

The similarity of signal peptides between the strains of the same and different species is shown for *L. plantarum* and *P. acidilactici* in Supplementary Fig. S2A and B, respectively. Both, *L. plantarum* and *P. acidilactici* showed a high similarity of predicted signal peptides across strains belonging to the same species. The average similarities (± standard deviation) across strains of *L. plantarum* and *P. acidilactici* were 51.1 ± 8.4% and 45.3 ± 14.6%, respectively. When comparing with strains of other species or even genera, the similarity of predicted signal peptides decreased, with a considerably steeper decrease noticed within the genus *Pediococcus* compared to the genus *Lactiplantibacillus*. For *Lactiplantibacillus*, the dendrogram derived from the signal peptide similarity matrix correlated well with the core genome phylogenetic tree [1].

## Discussion

Extensive research has been conducted on the optimization of the secretory expression of proteins in Lactobacillales [50, 51]. However, secretion of heterologous proteins remains challenging. Previously, most studies have focused on the identification and use of heterologous signal peptides [22, 52, 53]. Here, we present a newly designed vector as a tool for secretion of proteins from *Lactiplantibacillus plantarum* and *Pediococcus acidilactici*, followed by comprehensive secretory activity testing of clones with plasmids harboring one of a total of 155 and 110 homologous putative signal peptides predicted from the genomes of *L. plantarum* and *P. acidilactici*, respectively.

Previously, numerous plasmid vectors have been constructed for various lactobacilli [30–32, 54]. However, the selection of replicons for plasmid construction are host strain dependent. Thus, in this study, five different replicons were tested in order to develop a stable and advanced shuttle vector for protein expression in *L. plantarum* and *P. acidilactici*. Replication of bacterial plasmids by a rolling-circle mechanism such as described for pSH71 and pWV01 avoids bulkiness of the vector due to its broad host-range. However, in our application, the use of the rolling-circle mechanism resulted in instability of the plasmid structure. Similarly, earlier work suggested that the formation of linear high-molecular-weight plasmid multimers by rolling-circle replication was implicated in structure and segregational instability [55]. Therefore, we switched to a dual replicon strategy with both lactobacilli and *E. coli* replicons displayed in our vector. Moreover, to increase the ligation efficiency of the linearized backbone plasmid and DNA fragments of the signal peptide library, DNA assembly technology was used. Compared to the traditional digestion-ligation method, DNA assembly resulted in > 10 times higher ligation efficiency. Furthermore, to increase transformation efficiency, the *E. coli* strain C2925, was chosen for construction of the *L. plantarum* plasmid library in this study. So far, *E. coli* C2925 has been a rather neglected strain for cloning. However, its unmethylated plasmids were easily transformed into *L. plantarum* due to its type IV restriction modification (R-M) system. Understanding the R-M system of the ultimate host strain can be an effective strategy to increase its transformation efficiency.

The same expression system was exploited for all five proteases tested here, and codon optimization was performed for proteases in our host strains. However, only PepG1 could be expressed and secreted in both *L. plantarum* and *P. acidilactici*. The issue of no expression of the four remaining proteases could be due to i) a complex mRNA secondary structure preventing interactions with the host’s cellular machinery, leading to failed translation, ii) misfolding or unfolding due to lack of accurate post-translational modification, leading to fast degradation, or iii) toxicity of the protease due to its proteolytic activity when expressed inside the cell, and not secreted efficiently [56].

In this study, we used PepG1 as a model protein and studied secretion capacity with four typical heterologous signal peptides compared to homologous LP_0373. None of the heterologous signal peptides resulted in improved PepG1 secretion from strains *L. plantarum* and *P. acidilactici*. This result corroborates the consensus that a signal peptide’s secretion capacity is difficult to predict based on its sequence properties [27, 28]. Thus, the construction of homologous signal peptide libraries and high-throughput screening seem to be a necessary and promising approach to identify the optimal signal peptide for the target protein.

For *L. plantarum*, 12 out of 155 signal peptides were selected for highest PepG1 secretion. LP_23790, the best-performing signal peptide resulted in an 18% increase of protease activity in the culture media compared to LP_0373. Out of the 12 best-performing signal peptides, nine are novel signal peptides, among which LP_23670, LP_28190, LP_29340 and LP_14210 were previously undiscovered (less than 70% similarity), while LP_23790, LP_08330, LP_04240 and LP_24320 demonstrate one amino acid difference with other known *L. plantarum* signal peptides [27]. Furthermore, nine out of 12 signal peptides originate from proteins with unknown functions (cell surface protein, extracellular protein and hypothetical protein). It would be interesting to understand the function of these native secretory proteins in the future.

Even though several common heterologous signal peptides have been tested in *P. acidilactici* for protein secretion [54], to the best of our knowledge, this is the first study of genome-wide analysis of homologous signal peptides of *P. acidilactici*. PA_18600 showed the highest secretion capacity (80% higher than the control LP_0373). All eight signal peptides identified here from *P. acidilactici* are novel signal peptides. These signal peptides may also be promising candidates for the expression and secretion of other heterologous proteins in *P. acidilactici*.

The analysis of secretion capacity and sequence properties of respective signal peptide did not reveal any distinctive predictive properties. Therefore, the characteristics that make a suitable signal peptide for a particular protein remain to be elucidated at the molecular level. Even though some studies suggested that an increase of the positive charge within the N-domain and increased hydrophobicity of the H-domain could improve secretion in some bacteria [57], other studies delivered the opposite conclusion [58]. In consequence, instead of site-directed mutagenesis to purposely change signal peptide properties, e.g., charge, polarity and hydrophobicity, saturation mutagenesis may be a more promising strategy to modify the amino acid sequence in a saturated manner in future studies [59]. In addition, a few studies have shown that directed evolution of signal peptides can improve target protein secretion. This work involved the fusion of N-terminal signal peptide, target protein and C-terminal β-lactamase, and selection of best candidates by choosing the survival mutants after application of ampicillin as selection pressure [60, 61]. In addition, the secretion of a certain target protein is guarded by a complex pattern of events, involving a balance between biosynthesis, translocation and folding efficiency of the protein [46, 62]. For example, linearization of the mRNA secondary structure near the ribosome binding site was reported to increase secretory expression levels [57], and different propeptides were tested for improved secretion yield of endopeptidase in both *Lactococcus lactis* [41, 63] and *L. casei* [42]. Taking into account the above considerations, modification of the ribosome binding site and 5′ end mRNA sequence, and propeptide and promoter library screening can be exploited to further increase protease secretion in the future.

## Conclusions

In this study, plasmid, pUC256E, was developed for high-throughput screening of signal peptides in *L. plantarum* or *P. acidilactici*. Genome-wide experimental screening identified 20 signal peptides which show improved protease PepG1 secretion in either *L. plantarum* or *P. acidilactici*. The analysis of secretion capacity and sequence properties of respective signal peptide did not reveal any significant correlations. Therefore, it is not feasible to select the best-performing signal peptide for the target protein based on its amino acid sequence. The distribution of identified active signal peptides in other strains of the same species, and, in the case of *L. plantarum*, even in closely related species suggests their wider applicability. The genome-wide library screening approach presented in this study is an accessible and straightforward approach for high-throughput screening of signal peptides for the target protein.

## Methods

### Strains and growth conditions

*Escherichia coli* DH5α (Invitrogen, Carlsbad, USA) and *E. coli* C2925 (*dam*^*−*^*/dcm*^*−*^) (New England BioLabs, Ipswich, USA) cells were grown in LB (Lennox) broth (Bio basic, Toronto, Canada) at 37 °C under constant shaking. *Lactiplantibacillus plantarum* strain SH LP and *Pediococcus acidilactici* strain SH PA cells were grown stationary in MRS broth (Oxoid) with 0.1% Tween-80 (w/v) at 37 °C. Solid media were prepared with an addition of 1% agar (w/v) for LB plates, and 1.6% agar (w/v) for MRS plates. Antibiotics were added as follows: 100 μg/ml ampicillin for *E. coli*; 5 μg/ml erythromycin for *L. plantarum* and *P. acidilactici*.

### Plasmid construction

The cloning skeleton for the new (shuttle) vector can be found in Table 3. Plasmids were constructed using standard molecular cloning techniques. Primers used in this study were purchased from Integrated DNA Technologies (Supplementary Table S2). Plasmid pUC256E was designed as a shuttle vector to propagate in *E. coli*, *L. plantarum* and *P. acidilactici*. Plasmid pUC57, which contains backbone elements for plasmid propagation in *E. coli* was chosen as a starting vector. *E. coli* replicon pMB1 and ampicillin resistance marker (Amp^R^) were retained in pUC57. For propagation in *L. plantarum* and *P. acidilactici*, plasmid pUC256 was derived from pUC57 by ligating a lactobacilli replicon p256 [36, 64], synthesized from Bio Basic (Toronto, Canada), to the multiple cloning site (MCS). Erythromycin resistance marker (Erm^R^), and reporter gene β-glucuronidase (GusA) including lactobacilli phosphoglycerate mutase promoter (pgm promoter) and terminator (Term 908) were amplified from pTRK892m, an SaII mutated version of pTRK892 [32], and subsequently inserted into pUC256 using restriction sites NcoI and BglII, resulting in plasmid pUC256E. The 17 vectors that were generated in this study together with the plasmid library are shown in Table 3.

**Table 3:** Plasmids used in this study

Plasmids	Relevant characteristics	Source
pUC57	Amp^R^, pMB1 origin; cloning vector skeleton for shuttle vector	Bio Basic
pUC256	Amp^R^, pMB1 origin, p256 orgin	This study
pUC256E	Amp^R^, Erm^R^, pMB1 origin, p256 orgin, GusA reporter	This study
pUC256E-sp_LP_0373_-NprB	pUC256E carrying NprB fused to sp_LP_0373_ under P_pgm_ control	This study
pUC256E-sp_LP_0373_-pro-NprB	pUC256E carrying pro-NprB fused to sp_LP_0373_ under P_pgm_ control	This study
pUC256E-sp_LP_0373_-PepJ	pUC256E carrying PepJ fused to sp_LP_0373_ under P_pgm_ control	This study
pUC256E-sp_LP_0373_-pro-PepJ	pUC256E carrying pro-PepJ fused to sp_LP_0373_ under P_pgm_ control	This study
pUC256E-sp_LP_0373_-PepA-Aa	pUC256E carrying PepA-Aa fused to sp_LP_0373_ under P_pgm_ control	This study
pUC256E-sp_LP_0373_-pro-PepA-Aa	pUC256E carrying pro-PepA-Aa fused to sp_LP_0373_ under P_pgm_ control	This study
pUC256E-sp_LP_0373_-PepA-An	pUC256E carrying PepA-An fused to sp_LP_0373_ under P_pgm_ control	This study
pUC256E-sp_LP_0373_-pro-PepA-An	pUC256E carrying pro-PepA-An fused to sp_LP_0373_ under P_pgm_ control	This study
pUC256E-sp_LP_0373_-PepG1	pUC256E carrying PepG1 fused to sp_LP_0373_ under P_pgm_ control	This study
pUC256E-sp_LP_0373_-pro-PepG1	pUC256E carrying pro-PepG1 fused to sp_LP_0373_ under P_pgm_ control	This study
pUC256E-sp_LP_0600_-PepG1	pUC256E carrying PepG1 fused to sp_LP_0600_ under P_pgm_ control	This study
pUC256E-sp_sslipA_-PepG1	pUC256E carrying PepG1 fused to sp_sslipA_ under P_pgm_ control	This study
pUC256E-sp_M6_-PepG1	pUC256E carrying PepG1 fused to sp_M6_ under P_pgm_ control	This study
pUC256E-sp_Usp45_-PepG1	pUC256E carrying PepG1 fused to sp_Usp45_ under P_pgm_ control	This study
pUC256E-sp_LIBRARY_-PepG1	Plasmid library of pUC256E carrying PepG1 fused to homologous signal peptide under P_pgm_ control	This study

### Signal peptide cloning, assembly and transformation

Genomic DNA was extracted by using bead-beating in combination with the Maxwell DNA extraction system. Briefly, 150 μl of an overnight culture of *L. plantarum* or *P. acidilactici* were transferred into bead-beating tubes (MP Biomedicals LLC, Irvine, USA) together with 500 μl 1% SDS and 20 μl Protease K (Promega, Madison, USA). Bead-beating was performed at 6.0 m/s for 40 s using the FastPrep system (MP Biomedicals LLC, Irvine, USA). The sample was centrifuged at 16,000×g for 6 min. The supernatant was transferred into the first well of the Maxwell cartridge containing 300 μl of lysis buffer, and all subsequent steps were done as described in the manufacturer’s protocol (Maxwell 16 FFS Nucleic Acid Extraction System, Promega, Madison, USA). DNA was eluted into a total volume of 80 μl elution buffer (10 mM Tris, pH 8.5 with HCl). Whole genome sequencing was performed using Illumina NextSeq500 sequencing technology at Temasek Life Sciences Laboratory (Singapore). Sequence data was quality checked, and a draft genome was obtained after assembly using SPAdes [65]. The proteome profile was established by processing the genome through DDBJ Fast Annotation and Submission Tool (https://dfast.nig.ac.jp/help_annotation, [66]). Signal peptides and cleavage sites were predicted using the SignalP server (http://www.cbs.dtu.dk/services/SignalP/, [43]).

DNA sequences of signal peptides were amplified either from genomic DNA of target strains or through primer self-annealing using Phusion polymerase (New England BioLabs, Ipswich, USA). The PCR products were visually inspected for quality and size and then extracted from 2% DNA agarose gels using the QIAquick PCR Purification kit (Qiagen, Hilden, Germany). Subsequently, the various DNA fragments were combined at equal concentrations. The mixture together with the linearized vector pUC256E were assembled with 20 bp overlap using NEBuilder HiFi DNA Assembly Master Mix (New England BioLabs, Ipswich, USA). The assembled mixture was then transformed into *E. coli* DH5α and *E. coli* C2925 following the manufacturer’s protocol. After overnight growth, plasmids were extracted from transformed *E. coli* C2925 and *E. coli* DH5α cells, and immediately transformed into *L. plantarum* and *P. acidilactici*, respectively.

Cells of *L. plantarum* were transformed as described previously [67]. In brief, cell culture was re-inoculated into MRS broth with 1% glycine at an initial OD_600_ of 0.25. The cells were harvested at an OD_600_ of 0.5 and washed three times with an equal volume of 10 mM MgCl_2_, 1 mM MgCl_2_, and 30% (w/v) PEG 1500, in sequence. The cells were then resuspended in 400 μl of 30% (w/v) PEG 1500. A total of 100 μl of cells was mixed with plasmids in a 1 mm electrode-gap cuvette which received a single pulse from a Bio-Rad Xcell Gene Pulser at 2.5 kV, 25 μF and 400 Ω. Electroporated cells were recovered in MRS broth for 2 h at 37 °C. To identify the positive transformants, cells were spread on MRS plates with 5 μg/ml erythromycin and incubated for 2 days at 37 °C.

*P. acidilactici* was transformed based on the method previously described by Rodriguez et al. [68] with some modifications. In brief, overnight grown cells were re-inoculated into MRS broth with 40 mM DL-threonine at an initial OD_600_ of 0.25. The cells were harvested at an OD_600_ of 1.3 and washed three times with an equal volume of chilled electroporation buffer (0.6 M sucrose, 7 mM potassium phosphate, 1 mM MgCl_2_, pH 7.5). The cells were then incubated in pre-warmed lysozyme solution (2000 U/ml of cell suspension) for 25 min at 37 °C, harvested, washed three times and finally resuspended in 200 μl of electroporation buffer. Following the procedure of *L. plantarum*, a single pulse was added to the *P. acidilactici* and plasmid mixture with modified settings of 2.5 kV, 25 μF and 200 Ω. The electroporated cells were recovered and positive transformants selected as described for *L. plantarum* above.

### Protease activity assay

In this study, higher secretion capacity of the tested signal peptide was defined as a higher amount of protease secreted into the media [27]. Therefore, a protease activity assay was utilized to assess the influence of the different signal peptides on protease secretion. Freshly inoculated cultures of transformed *L. plantarum* or *P. acidilactici* were re-inoculated into fresh media in a 96-deep well plate at an initial OD_600_ of 0.1. After 24 h, the plates were centrifuged at 3000×g for 10 min, and protease activity in the supernatant was measured according to the EnzChek™ Protease Assay Kit (Invitrogen, Carlsbad, US). The assay is based on the detection of highly fluorescent BIODIPY FL dye-labeled peptides released by protease-catalyzed hydrolysis. Protease activities in the culture were expressed in fluorescence intensity units measured with an Infinite M Nano+ plate reader (Tecan, Zurich, Switzerland) with a filter fluorometer (excitation wavelength 485 nm, emission wavelength 530 nm). During the second round of screening, protease activity for each of the analyzed secretion tags was evaluated in triplicate.

### Western blot analysis

Cell pellet and culture supernatant were separated by centrifugation at 3000×g for 10 min. Proteins in the supernatant were precipitated with 100% (w/v) TCA at a final TCA concentration of 20%. The precipitate was washed with ice-cold acetone and centrifuged again at 20,000×g for 10 min. Intracellular proteins were released by lysing the cell pellets with a FastPrep homogenizer in lysis buffer (50 mM TrisHCl, 100 mM KCl, pH 7.9). Proteins precipitated from supernatants, and cell lysates were boiled with Laemmli sample buffer (Bio-Rad, Hercules, USA) and separated on 4-20% Mini-PROTEAN® TGX™ Precast Protein gels using TGX running buffer (Bio-Rad, Hercules, USA). The sample gels were used for blotting as described previously [69]. Proteins were blotted onto a 0.2 μm nitrocellulose membrane (Bio-Rad, Hercules, USA) through the Trans-Blot Turbo Blotting System (Bio-Rad, Hercules, USA). HRP conjugated anti-6× His-tag antibody (ThermoFisher Scientific, Waltham, USA) and Pierce ECL Western blotting substrate (Life Technologies, Carlsbad, USA) were used to detect 6× His-tagged proteins. The blotted membrane was visualized using the ChemiDoc system (Bio-Rad, Hercules, USA).

### Sequence analysis of signal peptide

The N-domain of signal peptide was defined as the peptide sequence starting from the N-terminal methionine up to the last positively charged amino acid [49]. The net charge of the N-domain was calculated with amino acid aspartic acid (D) and glutamic acid (E) defined as − 1, lysin (K) and arginine (R) defined as + 1 and all other amino acids as zero [70]. Hydrophobicity was calculated with amino acids glycine (G), alanine (A), valine (V), leucine (L), isoleucine (I), methionine (M), phenylalanine (F), tryptophan (W) and proline (P) defined as hydrophobic and the remaining defined as hydrophilic [70]. Previous studies have shown that signal peptides adopt α-helical conformations in interfacial environments such as cell membranes [27]. The transmembrane helix structure was predicted by a web-based transmembrane helical prediction program, TMHMM Server v. 2.0 (http://www.cbs.dtu.dk/services/TMHMM/, [71]).

### Signal peptides comparison within species and genera

To evaluate the relevance of our identified active signal peptides in other strains, all strains belonging to *L. plantarum* or *P. acidilactici*, for which complete genome sequences were available in NCBI (accessed on 10-Nov-2021), were downloaded. To further evaluate the pertinence in other *Lactiplantibacillus* or *Pediococcus* species, one representative strain per species, for which a complete genome sequences was available was chosen for analysis (complete genome sequences were not always available for the type strains). *L. plantarum* SN13T (GenBank assembly accession: GCA_013394345.1; failed taxonomy check in NCBI) was removed from the analysis. In total, 156 strains of *L. plantarum* and 23 strains of *P. acidilactici* were obtained. Seven species, namely, *L. argentoratensis*, *L. paraplantarum*, *L. pentosus*, *P. pentosaceus,* *P. claussenii*, *P. damnosus* and *P. inopinatus*, contained strains, for which complete genome sequences were available from NCBI, thus these representative strains were also included in the analysis. Signal peptides were predicted for all strains using SignalP. The presence of the identified active signal peptides in all strains was assessed using an in-house python script (available from the authors upon request).

To obtain insights into signal peptide similarity at species and genus level, signal peptides were predicted for all strains mentioned above using SignalP. Strains from *Secundilactobacillus malefermentans*, *Furfurilactobacillus rossiae*, *Lentilactobacillus buchneri* and *Levilactobacillus brevis* were used as an outgroup. An in-house python script was developed, which, first identified the shared signal peptides for each pair of strains, then generated a matrix with the numbers of shared signal peptides for all pairs of strains, and lastly converted the matrix to a new matrix providing the percentage of shared signal peptides over the total (unique and shared) signal peptides for all pairs of strains. The similarity heatmap was generated using ClustVis (https://biit.cs.ut.ee/clustvis/, [72]).

## Supplementary Information

**Additional file 1: Table S1.** Unique signal peptides identified from first round of screening and number of repetition in different clones.

**Additional file 2: Figure S1.** Full length image of western blots of *L. plantarum* and *P. acidilactici*. (A) Western blot image of *L. plantarum*. Lane 1-5: standard, *L. plantarum* wild type, *L. plantarum* with pUC256E, *L. plantarum* with pUC256E-sp_LP_0373_-PepG1, *L. plantarum* with pUC256E-sp_LP_0373_-pro-PepG1. (B) Western blot image of *P. acidilactici*. Lane 1-5: standard, *P. acidilactici* wild type, *P. acidilactici* with pUC256E, *P. acidilactici* with pUC256E-sp_LP_0373_-PepG1, *P. acidilactici* with pUC256E-sp_LP_0373_-pro-PepG1.

**Additional file 3: Table S2.** Primers used in this study. Restriction sites are in bold.

**Additional file 4: Figure S2.** Heatmap of signal peptide similarity comparison of *L. plantarum* (A) and *P. acidilactici* (B). GenBank assembly accession numbers are provided for each strain. Strains belonging to species other than *L. plantarum* and *P. acidilactici* are labelled with their species names and GenBank assembly accession numbers. Blue color indicates no similarity of predicted signal peptides between two strains, red color indicates 100% similarity of predicted signal peptides between two strains.

## Data Availability

All data generated or analysed during this study are included in this published article and its supplementary information files.
