# Supplementary figures and images for: Genome-wide high-throughput signal peptide screening via plasmid pUC256E improves protease secretion in Lactiplantibacillus plantarum and Pediococcus acidilactici
Source: BMC Genomics. 2022 Jan 12;23:48. doi: 10.1186/s12864-022-08292-3 (PMC8756648; doi:10.1186/s12864-022-08292-3)

Figure S1A

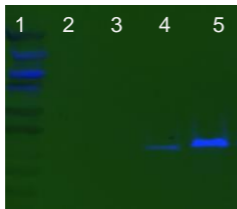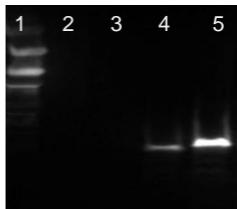

Figure S1B

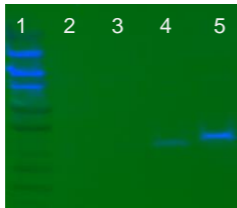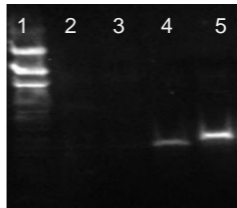

Supplement: Supplementary file 2 — Additional file 2: Figure S1. Full length image of western blots of L. plantarum and P. acidilactici. (A) Western blot image of L. plantarum. Lane 1-5: standard, L. plantarum wild type, L. plantarum with pUC256E, L. plantarum with pUC256E-spLP_0373-PepG1, L. plantarum with pUC256E-spLP_0373-pro-PepG1. (B) Western blot image of P. acidilactici. Lane 1-5: standard, P. acidilactici wild type, P. acidilactici with pUC256E, P. acidilactici with pUC256E-spLP_0373-PepG1, P. acidilactici with pUC256E-spLP_0373-pro-PepG1. [file 12864_2022_8292_MOESM2_ESM.pdf]

Figure S2A

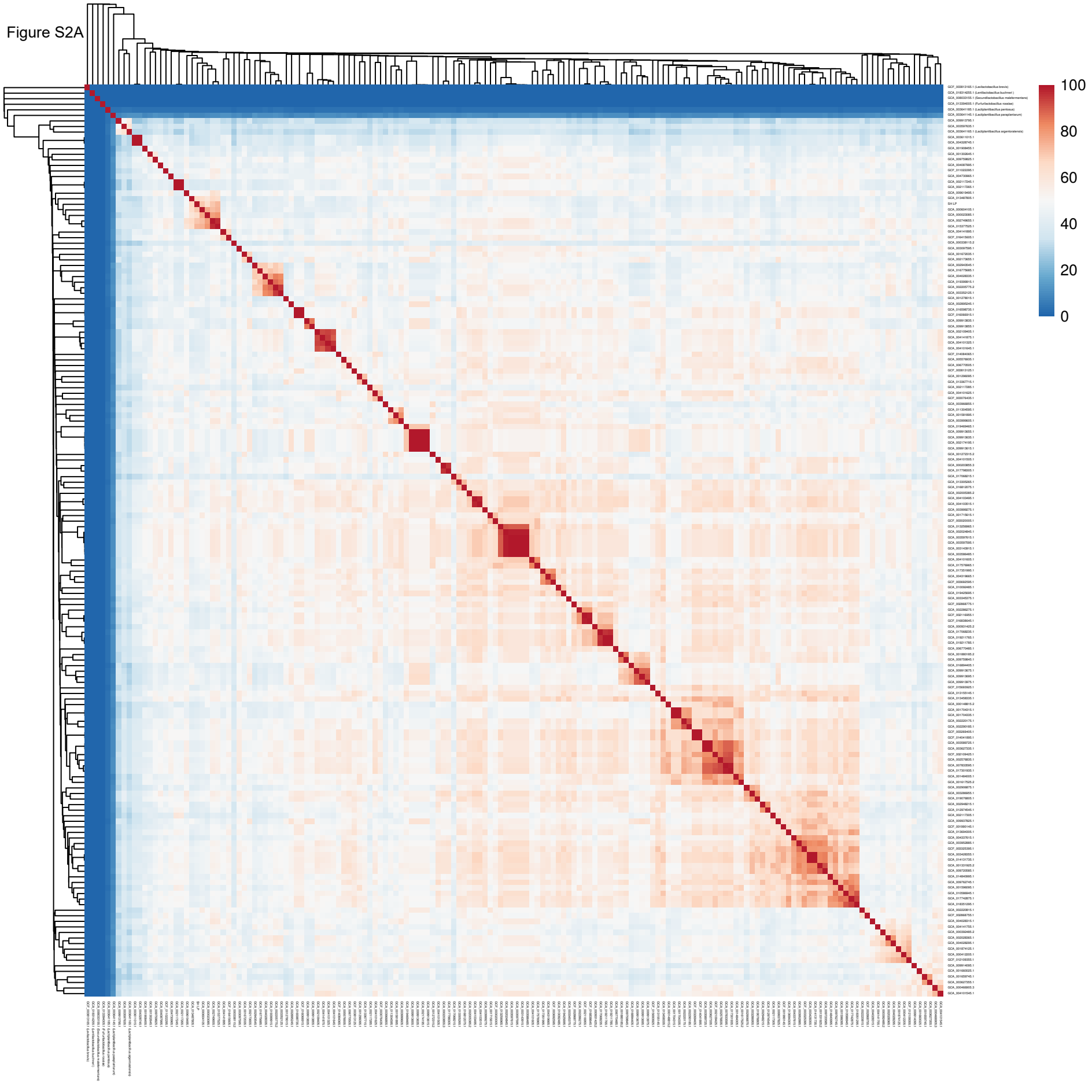

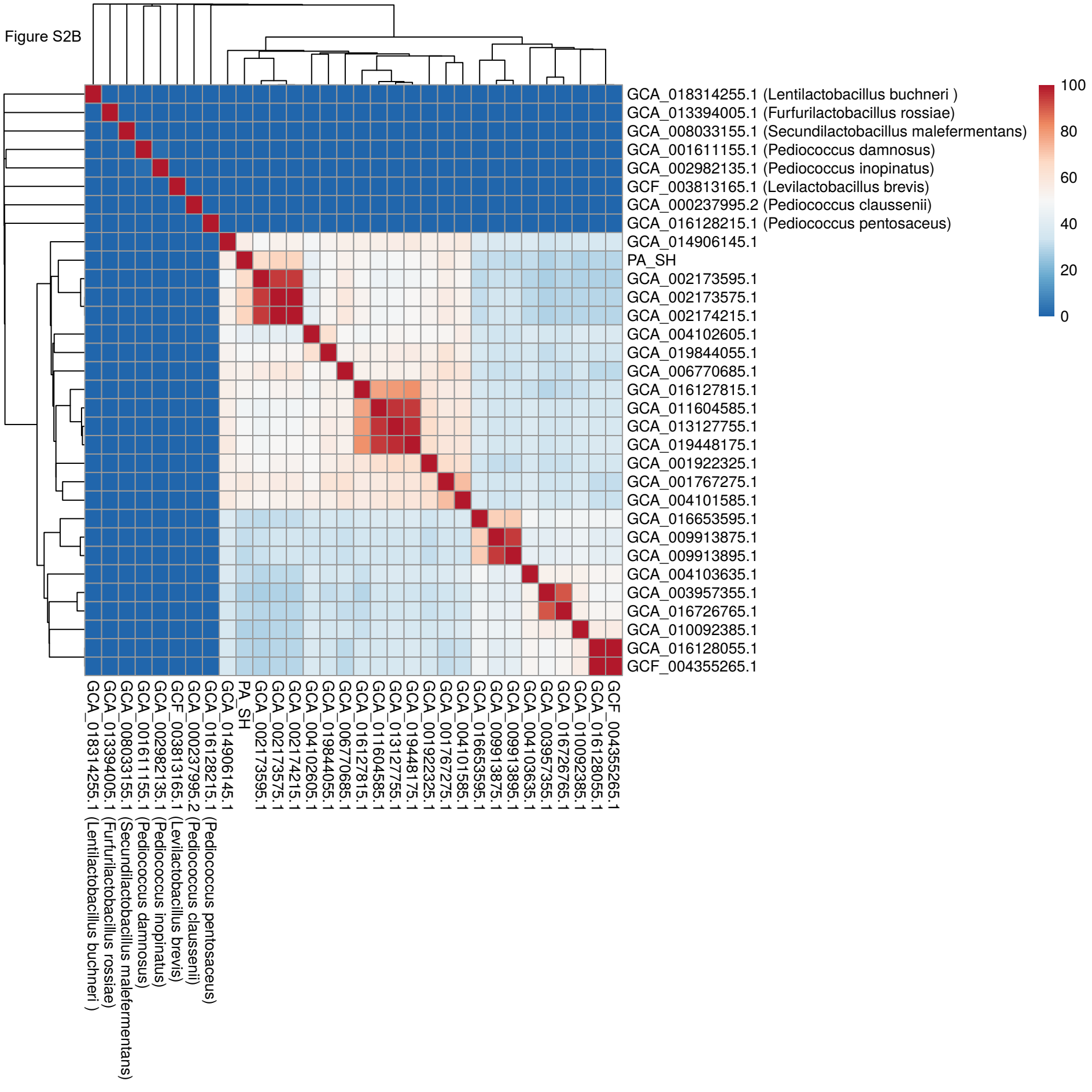

Supplement: Supplementary file 4 — Additional file 4: Figure S2. Heatmap of signal peptide similarity comparison of L. plantarum (A) and P. acidilactici (B). GenBank assembly accession numbers are provided for each strain. Strains belonging to species other than L. plantarum and P. acidilactici are labelled with their species names and GenBank assembly accession numbers. Blue color indicates no similarity of predicted signal peptides between two strains, red color indicates 100% similarity of predicted signal peptides between two strains. [file 12864_2022_8292_MOESM4_ESM.pdf]
